# Liver-related outcomes in patients with cirrhosis: The value of clinical and laboratory data and noninvasive tests

**DOI:** 10.1371/journal.pone.0326702

**Published:** 2025-07-01

**Authors:** Somaya Albhaisi, Amanda Robinson, Rasha Alsaadawi, Roy T. Sabo, Arun J. Sanyal

**Affiliations:** 1 Department of Internal Medicine, Virginia Commonwealth University, Richmond, Virginia, United States of America; 2 C. Kenneth and Dianne Wright Center for Clinical and Translational Research, Virginia Commonwealth University, Richmond, Virginia, United States of America; 3 Department of Biostatistics, School of Population Health, Virginia Commonwealth University, Richmond, Virginia, United States of America; 4 Division of Gastroenterology, Hepatology and Nutrition, Department of Internal Medicine, Virginia Commonwealth University, Richmond, Virginia, United States of America; University of Navarra School of Medicine and Center for Applied Medical Research (CIMA), SPAIN

## Abstract

**Background and Purpose:**

Cirrhosis patients face high mortality risks from complications, emphasizing the need for prognostic tools to estimate risk of adverse liver outcomes based on cirrhosis etiology and hence improve disease management. This study aimed to evaluate the association between baseline clinical factors, lab values and noninvasive measurements and risk of adverse liver outcomes among patients with cirrhosis, and develop disease-specific associative models.

**Methods:**

Using proportional hazards regression, we developed six associative models, categorized by cirrhosis etiology, after identifying significant predictors of 30-day risk of ascites, hepatic encephalopathy (HE), and variceal bleeding (VB) among cirrhosis patients, with measurements selected through LASSO regression.

**Results:**

A total of 4045 adult patients with cirrhosis were included in the analysis from a single U.S. center retrospective cohort. The 5-year rates for ascites, HE, and VB were 31.6%, 22.9%, and 30.7%, respectively. Multivariable analyses showed that independent predictors in cirrhosis due to metabolic dysfunction-associated steatohepatitis (MASH) and viral hepatitis were: (a) ascites: albumin and international normalized ratio (INR); (b) HE: albumin, INR, total bilirubin, platelet count; (c) VB: albumin, platelet count, hemoglobin. No variables were significantly associated with outcomes in patients with alcohol-associated liver disease.

**Conclusions:**

The newly developed models provided accurate estimates of the 30-day risks of ascites, HE, and VB in MASH or viral hepatitis-related cirrhosis using baseline variables, providing a reliable tool for identifying high-risk patients warranting intensified interventions.

## 1. Introduction

Cirrhosis is the most advanced stage of chronic liver disease with a progressive clinical course. It is associated with high morbidity and mortality and extensive utilization of health care resources [1,2]. Treatment options for patients with cirrhosis are quite complex due to cirrhosis burden and outcomes. For healthcare providers, it is important to be able to predict risk of hepatic decompensation in patients with cirrhosis to optimize their care, provide counseling and improve preventive strategies. The ability to detect the patients at greatest risk of liver complications among the vast number of patients with cirrhosis is a key issue. The most commonly utilized tools to predict outcomes in patients with cirrhosis include hepatic venous pressure gradient measurement, Model for End-stage Liver Disease (MELD) and Child–Pugh–Turcotte (CPT) scores, and clinical staging systems [2]. The Fibrosis-4 index (FIB-4), using age, aspartate aminotransferase (AST), alanine aminotransferase (ALT), and platelet count, is a noninvasive tool that effectively identifies advanced fibrosis in metabolic dysfunction-associated steatotic liver disease (MASLD) patients and has been shown to have a prognostic value for prediction of adverse outcomes [3,4]. Predicting clinical outcomes in patients with cirrhosis remains challenging, and is likely best achieved with a combination of clinical information and objective data using lab values and noninvasive tests (such as MELD and FIB-4). It remains unclear, however, whether the use of noninvasive tests like FIB-4 index in routine clinical practice can identify the subgroup of people with cirrhosis at higher risk for development of liver-related complications. In addition, it is not clear whether the cut-off values for noninvasive biomarkers used to estimate advanced fibrosis and cirrhosis are optimal for the prediction of clinical outcomes. Predicting, or at the very least, estimating the risk of clinical outcomes in patients with liver cirrhosis is essential for risk stratification, long term monitoring of disease progression, prevention of complications, transplant evaluation and improving patient care. Healthcare providers can benefit from using simple tools to evaluate the association between baseline clinical and lab variables, and estimate risk of adverse outcomes to identify patients at high risk for development of complications, thereby providing a clinical decision tool that can help improve patient care and treatment outcomes. Advanced statistical methods, combined with robust clinical data, enable the development of reliable associative models that can be integrated into clinical practice to manage adverse outcomes proactively. While existing tools such as the MELD and CPT scores are valuable for predicting mortality in cirrhosis, they are less effective in estimating short-term risks of complications such as ascites, HE, and VB. This gap underscores the need for simpler, noninvasive models that leverage routinely available clinical and laboratory data to identify high-risk patients early. Our study addresses this need by developing and validating associative models tailored to specific etiologies of cirrhosis, providing a practical tool for clinicians to optimize patient care and preventive strategies. This study aims to develop and validate etiology-specific associative models for predicting 30-day risk of adverse liver-related outcomes (ascites, HE, VB) in cirrhosis patients using baseline clinical and laboratory variables. The primary objective is to create accurate, noninvasive tools for short-term risk stratification. Secondary objectives include evaluating the prognostic value of noninvasive biomarkers, identifying etiology-specific predictors, and assessing the feasibility of integrating these models into clinical practice. We hypothesize that baseline variables can be used to develop etiology-specific models that accurately predict short-term complications in cirrhosis patients. Specifically, we expect noninvasive biomarkers to have significant prognostic value independent of traditional scoring systems. Additionally, we hypothesize that etiology-specific models may provide more accurate risk stratification than generalized models, particularly for patients with MASLD and viral hepatitis, and that these models will improve early identification of high-risk patients in routine clinical practice.

## 2. Patients and methods

### Study cohort and data collection

In this retrospective cohort study, we identified all adult patients (age > 18 years) with cirrhosis diagnosis who received care in any clinical setting (inpatient or outpatient) at Virginia Commonwealth University Health System (VCUHS) between January 2005 and December 2015; defined by having at least one inpatient or outpatient visit for any indication during the study period. A total of 4045 patients with diagnosis of cirrhosis due to alcohol-associated liver disease (ALD), viral hepatitis, or nonalcoholic steatohepatitis (NASH) (now known as metabolic dysfunction-associated steatohepatitis (MASH)) were included in the analysis. We identified patients who had an outcome diagnosis of interest on or after index date. We derived data on all patients with cirrhosis from electronic health records (CERNER). Patients with cirrhosis were identified using International Classification of Diseases (ICD) 9 and 10 diagnosis codes (supplementary table 1) and billing data. To enhance the reliability of our cohort, we applied strict inclusion criteria, such as requiring multiple instances of cirrhosis-related ICD codes and excluding patients with inconsistent or unsupported diagnoses. We did not exclude patients with pre-existing decompensation outcomes (HE, ascites, and VB) at the index date. This design choice reflects real-world clinical scenarios and allows our associative models to be applicable to a broad patient population, including those at various stages of disease progression. Baseline variables were measured at the index date, preceding the outcomes of interest during follow-up, to minimize concerns about reverse causality. Data extracted from patients’ records included demographic data (age, sex, race, ethnicity), smoking status, lab tests (complete blood count, white blood cell, hemoglobin, platelet counts), hepatic panel (ALT, AST, total bilirubin, direct bilirubin, albumin, and international normalized ratio (INR)), kidney function (creatinine, glomerular filtration rate (GFR)), lipid panel (total cholesterol, low-density lipoprotein (LDL), high-density lipoprotein (HDL), triglycerides), hemoglobin A1C (HbA1C)), comorbidities (type 2 diabetes mellitus (T2DM), hypertension, coronary artery disease, heart failure, sleep apnea, other cancers), and vital signs.

#### Definition of cirrhosis.

Diagnosis of cirrhosis was established based on the report of associated ICD-9 and ICD-10 codes, recorded at least once in any inpatient or outpatient encounter. The ICD codes that we used for cirrhosis and complications of cirrhosis are given in supplementary table 1. The top three etiologies of cirrhosis in our study population were MASLD, viral hepatitis and ALD.

1. MASLD/MASH: The diagnosis of MASH cirrhosis was based on the presence of ICD 9 and 10 codes for cirrhosis due to MASLD or MASH (571.8, 571.9, K75.81, K76.0, 571.5), recorded at least once in any inpatient or outpatient encounter, in absence of alcohol use disorders and ICD diagnostic codes for other etiologies of cirrhosis including viral, alcoholic, cholestatic, autoimmune or genetic.
2. Viral hepatitis: We identified patients with chronic hepatitis B and C based on ICD 9 and 10 codes for hepatitis B (070.22, B18. 1) or hepatitis C (070.54, B18. 2) regardless of any additional etiologies or treatment status.
3. ALD: Patients with ICD 9 and 10 codes for ALD (571.0, 571.1, 571.2, 571.3, K70.10-11) and alcohol use disorder (F10), regardless of any additional etiologies, were categorized as ALD.

Patients with other etiologies of cirrhosis were not included in the study because their sample sizes were too small.

#### Clinical characteristics.

The patients’ baseline characteristics were ascertained at the index visit (first encounter) when a diagnosis of cirrhosis was first recorded. Demographic data, lab tests and comorbidities were extracted from patients’ records. We obtained results closest to index date (±3 months) for patients who had multiple lab tests or measurements. Comorbidities were also identified using ICD-9 and 10 codes. Body mass index (BMI) was calculated as weight in kilograms divided by the square of height in meters. Patients were classified according to their BMI as follows: underweight (BMI < 18.5 kg/m2), normal weight (BMI 18.5–24.9 kg/m2), overweight (BMI 25–29.9 kg/m2), and obese (BMI ≥ 30 kg/m2). FIB-4 index was calculated using age, AST (U/L), ALT (U/L), and platelet count (10^9^/L), using standard formula [5]. MELD score was calculated using the standard formula (MELD = 9.57*ln(Creatinine)+3.78*ln(Total Bilirubin)+11.2*ln(INR)+6.43 [6].

#### Outcomes of interest.

We assessed the development of the following liver-related outcomes during follow-up: ascites, HE, or VB. These outcomes were defined based on ICD-9 and ICD-10 codes (supplementary table 1) that appeared at least once during the study follow up. Patients were retrospectively followed until the end of September, 2021, for the development of these outcomes.

### Statistical analysis

Descriptive statistics were calculated to investigate study population’s characteristics. Continuous variables were summarized with median and range, while categorical variables were summarized with frequency and percentage (Table 1). We used multiple imputation with 16 iterations to estimate missing values for covariates, with separate imputations used for each of model selection, validation, and model fitting [7]. We used proportional hazards LASSO regression to identify which measures to include in modeling as well as their functional forms (Table 2). Tuning parameters were selected by minimizing model deviance using complete case data with 5-fold cross validation. Polynomials up to the fifth power for each continuous measure were included to capture the nonlinearity discovered during diagnostics. Models were then fit for each iteration of the imputed data set with the same tuning parameter, and coefficients were averaged across imputations. Terms with non-zero averaged coefficients were included in the model. Continuous measures with non-zero coefficients for any polynomial term were included as restricted cubic splines with knots at the 10th, 50th, and 90th percentiles. We validated each model using five-fold cross validation. Thirty-day cumulative incidence was calculated from semi-parametric proportional hazard regression models for the testing data and averaged across imputation models. For model validation, we used the obtained models to predict the 30-day cumulative incidence for the testing data and compare it with the observed outcome. For each model, a summary of sensitivity, specificity, positive predictive value (PPV), and negative predictive value (NPV) was reported, where Youden Index was used to specify the optimal cutoff for identifying predicted “events” (Table 3). After validation, we refit the semi-parametric proportional hazards regression models with all records (Table 4). The log-hazard coefficients, standard errors, hazard ratios (HR), 95% confidence intervals (CI) and p-values were used to statistically test 30-day risk of adverse outcomes. A p-value <0.05 was considered as statistically significant. All statistical analyses were performed using R 4.2.1. and R 4.2.3. software.

**Table 1:** Demographic and clinical measures for cirrhosis patients by disease group.

	Alcohol-Associated Liver Disease (N = 578)	Viral Hepatitis (N = 2075)	Metabolic dysfunction-associated steatohepatitis (N = 1338)	Overall^*^ (N = 4045)
**Age At Index Date (years)**				
Median [Min, Max]	53.0 [23.0, 83.0]	53.0 [18.0, 87.0]	57.0 [18.0, 87.0]	54.0 [18.0, 87.0]
**Sex**				
Female	217 (37.5%)	788 (38.0%)	764 (57.1%)	1858 (45.9%)
Male	361 (62.5%)	1287 (62.0%)	574 (42.9%)	2187 (54.1%)
**Race**				
White	396 (68.5%)	1134 (54.7%)	1061 (79.3%)	2656 (65.7%)
Black Or African American	158 (27.3%)	846 (40.8%)	218 (16.3%)	1209 (29.9%)
Other	13 (2.2%)	43 (2.1%)	40 (3.0%)	99 (2.4%)
Asian	1 (0.2%)	36 (1.7%)	7 (0.5%)	46 (1.1%)
American Indian-Alaskan	0 (0%)	6 (0.3%)	1 (0.1%)	7 (0.2%)
Unknown-Unable To Communicate	2 (0.3%)	1 (0.0%)	3 (0.2%)	6 (0.1%)
Unknown-Patient Refusal	1 (0.2%)	1 (0.0%)	2 (0.1%)	4 (0.1%)
Multiple	2 (0.3%)	2 (0.1%)	0 (0%)	3 (0.1%)
Native Hawaii/Other Pac Island	1 (0.2%)	1 (0.0%)	0 (0%)	1 (0.0%)
Missing	4 (0.7%)	5 (0.2%)	6 (0.4%)	14 (0.3%)
**Ethnicity**				
Not Hispanic or Latino or Spanish Origin	483 (83.6%)	1790 (86.3%)	1120 (83.7%)	3440 (85.0%)
Hispanic or Latino or Spanish Origin	9 (1.6%)	40 (1.9%)	36 (2.7%)	87 (2.2%)
Unknown – Unable to Communicate	3 (0.5%)	5 (0.2%)	7 (0.5%)	15 (0.4%)
Missing	83 (14.4%)	240 (11.6%)	173 (12.9%)	501 (12.4%)
**BMI (Kg/m**^**2**^)				
Median [Min, Max]	27.0 [10.5, 56.7]	28.3 [11.4, 186]	31.9 [9.69, 310]	29.2 [7.03, 642]
Missing	85 (14.7%)	554 (26.7%)	432 (32.3%)	1108 (27.4%)
**Diabetes Type 2**				
No Diabetes	458 (79.2%)	1645 (79.3%)	987 (73.8%)	3125 (77.3%)
Diabetes	120 (20.8%)	430 (20.7%)	351 (26.2%)	920 (22.7%)
**Hemoglobin Level (g/dL)**				
Median [Min, Max]	11.4 [3.80, 18.5]	13.8 [5.40, 18.7]	12.6 [4.90, 17.8]	13.1 [3.80, 19.7]
Missing	9 (1.6%)	87 (4.2%)	121 (9.0%)	226 (5.6%)
**White Blood Cell Count (x10**^**3**^**/mm**^**3**^)				
Median [Min, Max]	6.50 [0.600, 95.8]	6.10 [0.700, 54.6]	6.40 [0.300, 35.4]	6.20 [0.300, 95.8]
Missing	9 (1.6%)	88 (4.2%)	125 (9.3%)	231 (5.7%)
**Platelet Count (x10** ^**9**^ **/L)**				
Median [Min, Max]	126 [5.00, 767]	166 [5.00, 943]	161 [8.00, 894]	163 [5.00, 943]
Missing	9 (1.6%)	88 (4.2%)	121 (9.0%)	227 (5.6%)
**Aspartate Aminotransferase (U/L)**				
Median [Min, Max]	67.0 [11.0, 3600]	67.0 [9.00, 1280]	50.0 [9.00, 4080]	59.0 [9.00, 4080]
Missing	11 (1.9%)	106 (5.1%)	123 (9.2%)	247 (6.1%)
**Alanine Transaminase (U/L)**				
Median [Min, Max]	39.0 [5.00, 3460]	65.0 [5.00, 1770]	42.0 [5.00, 7220]	52.0 [5.00, 7220]
Missing	11 (1.9%)	105 (5.1%)	123 (9.2%)	246 (6.1%)
**FIB-4**				
Median [Min, Max]	4.61 [0.327, 92.5]	2.77 [0.355, 92.5]	3.01 [0.166, 69.5]	2.99 [0.166, 92.5]
Missing	13 (2.2%)	119 (5.7%)	145 (10.8%)	287 (7.1%)
**Total Bilirubin (mg/dL)**				
Median [Min, Max]	1.90 [0.100, 43.0]	0.700 [0.100, 48.2]	0.800 [0.100, 46.9]	0.800 [0.100, 48.2]
Missing	14 (2.4%)	115 (5.5%)	128 (9.6%)	264 (6.5%)
**Albumin Level (g/dL)**				
Median [Min, Max]	3.20 [1.50, 5.30]	4.00 [1.20, 5.30]	3.80 [1.20, 5.30]	3.90 [1.20, 5.30]
Missing	13 (2.2%)	110 (5.3%)	126 (9.4%)	256 (6.3%)
**International Normalized Ratio**				
Median [Min, Max]	1.30 [0.900, 4.20]	1.10 [0.800, 4.50]	1.10 [0.900, 5.80]	1.10 [0.800, 5.80]
Missing	30 (5.2%)	188 (9.1%)	156 (11.7%)	385 (9.5%)
**Creatinine Level (mg/dL)**				
Median [Min, Max]	0.820 [0.220, 8.18]	0.820 [0.270, 18.2]	0.820 [0.180, 18.7]	0.820 [0.180, 18.7]
Missing	12 (2.1%)	204 (9.8%)	150 (11.2%)	386 (9.5%)
**MELD**				
Median [Min, Max]	10.9 [1.00, 46.4]	4.80 [1.00, 45.4]	6.28 [1.00, 44.3]	5.77 [1.00, 46.4]
Missing	42 (7.3%)	321 (15.5%)	221 (16.5%)	599 (14.8%)
**Ascites**				
No	254 (43.9%)	1600 (77.1%)	840 (62.8%)	2766 (68.4%)
Yes	324 (56.1%)	475 (22.9%)	498 (37.2%)	1279 (31.6%)
**Hepatic Encephalopathy**				
No	327 (56.6%)	1745 (84.1%)	969 (72.4%)	3118 (77.1%)
Yes	251 (43.4%)	330 (15.9%)	369 (27.6%)	927 (22.9%)
**Variceal Bleeding**				
No	291 (50.3%)	1480 (71.3%)	918 (68.6%)	2803 (69.3%)
Yes	287 (49.7%)	595 (28.7%)	420 (31.4%)	1242 (30.7%)
**Ascites Follow-Up (days)**				
Median [Min, Max]	134 [0, 1830]	1640 [0, 1830]	581 [0, 1830]	1040 [0, 1830]
**Encephalopathy Follow-Up (days)**				
Median [Min, Max]	294 [0, 1830]	1830 [0, 1830]	829 [0, 1830]	1240 [0, 1830]
**Variceal Bleeding Follow-Up (days)**				
Median [Min, Max]	227 [0, 1830]	1200 [0, 1830]	568 [0, 1830]	855 [0, 1830]

* Note that the subtotals do not sum to the overall total because patients could be diagnosed with both alcohol-associated liver disease and viral hepatitis.

**Table 2:** LASSO model selection formulas by outcome and disease group.

Disease Group	Outcome	Formula
**Alcohol-Associated Liver Disease**	Ascites	~ 1
	Encephalopathy	~ 1
	Variceal Bleeding	~ 1
**Viral Hepatitis**	Ascites	~ rcs(albumin, c(2.6, 3.9, 4.5))
	Encephalopathy	~ platelet + rcs(albumin, c(2.6, 3.9, 4.5)) + inr + total bilirubin
	Variceal Bleeding	~ platelet + rcs(albumin, c(2.6, 3.9, 4.5)) + hemoglobin
**Metabolic dysfunction-associated steatohepatitis**	Ascites	~ albumin + inr
	Encephalopathy	~ rcs(albumin, c(2.6, 3.9, 4.5)) + inr
	Variceal Bleeding	~ platelet + albumin

rcs, restricted cubic splines with knots at the 10th, 50th, and 90th percentiles.

**Table 3:** Model validation results.

Disease Group	Outcome	Sensitivity	Specificity	Positive Predictive Value	Negative Predictive Value	AUC
**Metabolic dysfunction-associated steatohepatitis**	Ascites	0.66	0.65	0.29	0.90	0.65
	Encephalopathy	0.80	0.62	0.18	0.97	0.71
	Variceal Bleeding	0.07	0.80	0.03	0.90	0.43
**Viral Hepatitis**	Ascites	0.90	0.68	0.19	0.99	0.79
	Encephalopathy	0.78	0.72	0.07	0.99	0.75
	Variceal Bleeding	0.53	0.75	0.13	0.96	0.64

AUC, area under the curve

**Table 4:** Proportional hazard regression results by disease group and outcome.

Disease Group	Outcome	Variable	Estimate	Standard Error	Hazard Ratio [95% CI]	p-value
**Metabolic dysfunction-associated steatohepatitis**	Ascites	Albumin	−1.1227	0.0435	0.33[0.30, 0.35]	<0.0001
		INR	0.6997	0.0791	2.01[1.72, 2.35]	<0.0001
	Encephalopathy	Albumin	−0.3811	0.0925	0.68[0.57, 0.82]	<0.0001
		Nonlinear Albumin	−1.1567	0.1459	0.31[0.24, 0.42]	<0.0001
		INR	1.0993	0.0847	3.00[2.54, 3.54]	<0.0001
	Variceal Bleeding	Platelet Count (10 unit increase)	−0.0466	0.0040	0.95[0.95, 0.96]	<0.0001
		Albumin	−0.6597	0.0404	0.52[0.48, 0.56]	<0.0001
**Viral Hepatitis**	Ascites	Albumin	−0.7861	0.0717	0.46[0.40, 0.52]	<0.0001
		Nonlinear Albumin	−1.0223	0.1184	0.36[0.29, 0.45]	<0.0001
	Encephalopathy	Platelet Count (10 unit increase)	−0.0286	0.0044	0.97[0.96, 0.98]	<0.0001
		Albumin	−0.3202	0.0952	0.73[0.60, 0.87]	0.0004
		Nonlinear Albumin	−1.0635	0.1476	0.35[0.26, 0.46]	<0.0001
		INR	0.8387	0.1003	2.31[1.90, 2.82]	<0.0001
		Total Bilirubin	0.0431	0.0086	1.04[1.03, 1.06]	<0.0001
	Variceal Bleeding	Platelet Count (10 unit increase)	−0.0449	0.0040	0.96[0.95, 0.96]	<0.0001
		Albumin	−0.1837	0.0836	0.83[0.71, 0.98]	0.0140
		Nonlinear Albumin	−0.4793	0.1010	0.62[0.51, 0.75]	<0.0001
		Hemoglobin	−0.0898	0.0153	0.91[0.89, 0.94]	<0.0001

### Ethical statements

Study researchers made the determination that this study does not constitute human subject research given that the study uses secondary de-identified data [8].

## 3. Results

### Patient characteristics and outcomes

Demographics and baseline clinical characteristics of the overall patient population and by disease group are summarized in Table 1. We included 4045 adult patients with cirrhosis in the study. Of these, 578 were diagnosed with ALD, 2075 were diagnosed with viral hepatitis, and 1338 were diagnosed with MASH. The overall median age was 54 years, 53 years for patients with ALD and viral hepatitis, and 57 years for patients with MASH. Overall, 54% of patients were male, compared to 63% of patients with ALD, 62% of patients with viral hepatitis, and 43% of MASH patients. Overall, median BMI was 29 kg/m^2^, 27 kg/m^2^ for patients with ALD, 28 kg/m^2^ for patients with viral hepatitis, and 32 kg/m^2^ for MASH patients. Roughly, 23% of patients overall were diagnosed with diabetes, compared to 21% of patients with ALD or viral hepatitis and 32% of MASH patients. The majority of patients overall were non-Hispanic white (66%), and patients with viral hepatitis were less often white (55%) compared to patients with ALD (69%) or MASH (79%). Black and African American patients accounted for 30% of patients overall, 27% of patients with ALD, and 16% of MASH patients. The overall 5-year rates were 31.6%, 22.9%, and 30.7% for ascites, HE, and VB, respectively.

### Factors associated with 30-day risk of liver-related outcomes

**Fig 1:**
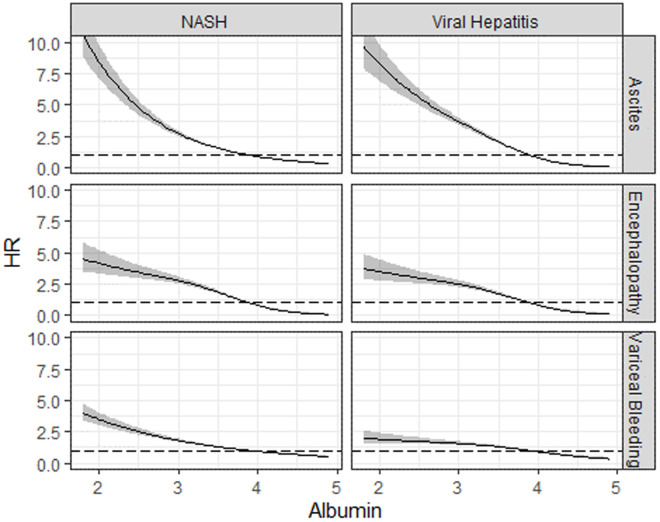
Hazard Ratios and 95% Confidence Intervals Relative to Median by Albumin, Disease Group, and Outcome.

We found that reduced albumin level was associated with increased risk of all outcomes in cirrhosis due to MASH and viral hepatitis (Fig 1). Increased INR was associated with increased risk of ascites in MASH cirrhosis patients (HR: 2.0, 95% CI: 1.7–2.4) and HE in both disease groups (HR: 3.0, 95% CI: 2.5–3.5 in MASH; HR: 2.3, 95% CI: 1.9–2.8 in viral hepatitis). There was a 5% decrease in risk of VB for each unit increase in platelet count in cirrhosis due to MASH (HR: 0.95, 95% CI: 0.95–0.96); and viral hepatitis (HR: 0.96, 95% CI: 0.95–0.96). Similarly, there was a 3% decrease in risk of HE for each unit increase in platelet count in patients with viral hepatitis (HR: 0.97, 95% CI: 0.96–0.98). Increased total bilirubin was associated with increased risk of HE in patients with viral hepatitis (HR: 1.04, 95% CI: 1.03–1.06). Increased hemoglobin was associated with decreased risk of VB in patients with viral hepatitis (HR: 0.91, 95% CI: 0.89–0.94). None of the variables were associated with outcomes in ALD cirrhosis.

### Model generation and validation

Cross-validation datasets were used to evaluate the validity of our associative models for 30-day risk of adverse liver-related outcomes, the summaries for which are presented in Tables 2–4. For the MASH disease group, we see that the model has satisfactory predictability for ascites (sensitivity = 0.66; specificity = 0.65), very good predictability for HE (sensitivity = 0.80; specificity = 0.62), but mixed results for VB (sensitivity = 0.07; specificity = 0.80). For the viral hepatitis disease group, the fitted model has good predictability for both Ascites (sensitivity = 0.90; specificity = 0.68) and HE (sensitivity = 0.78; specificity = 0.72), though it was less effective in predicting VB (sensitivity = 0.53; specificity = 0.73). While our models show capability for estimating 30-day risks, no one set of covariates fits all outcomes and disease groups. Albumin was nonlinearly associated with ascites in patients with viral hepatitis. Platelet count, nonlinear albumin, INR, and total bilirubin were associated with HE in patients with viral hepatitis. Platelet count, nonlinear albumin, and hemoglobin were associated with VB in patients with viral hepatitis. Albumin and INR were associated with ascites in MASH patients. Nonlinear albumin and INR were associated with HE in MASH patients. Platelet count and albumin were associated with VB in MASH patients. Next, the testing data was used for model validation and results are summarized in Tables 3–4.

## 4. Discussion

The results of our study show that models combining baseline variables that include albumin, INR, and platelet count were strongly associated with decompensation events and could estimate 30-day risk of adverse liver-related outcomes in patients with cirrhosis due to MASH or viral hepatitis. Cross-validation analysis of the models demonstrated the models to be reasonably predictive with a high calibration and a very good discrimination of cirrhosis patients at high risk for complications. We conducted this study on a large cohort of cirrhosis patients over a 5-year period. Our new simplified models accurately and consistently estimated the 30-day risk of adverse liver-related outcomes among patients with cirrhosis. Most existing scoring systems such as MELD score and CPT have been developed to predict mortality in cirrhosis patients rather than risk of developing other cirrhosis complications (ascites, HE, VB). Therefore, we developed the new models to address the need for noninvasive tools for early identification of high-risk cirrhosis patients to guide management and improve clinical care. We used the time frame of 30 days for assessing the short-term risk of adverse cirrhosis outcomes in a patient population that had a documented diagnosis of cirrhosis at baseline. While our models focus on short-term risk prediction, longer-term models may be more appropriate for patients with compensated cirrhosis. Future studies could explore the development of models with extended follow-up periods to better capture the risk of first decompensation.

Our model for assessing VB risk in MASH cirrhosis had high specificity but poor sensitivity due to the focus on maximizing the Youden Index. This approach often favors high sensitivity at the expense of specificity. Despite this, the model had a high NPV, likely due to its high specificity compensating for lower sensitivity. Our model for assessing VB risk in viral hepatitis cirrhosis had high specificity but low PPV. High specificity helps PPV, but in low-prevalence settings, even a few false positives can greatly reduce PPV. The lack of association between study variables and adverse outcomes in ALD cirrhosis may be due to unaccounted confounding factors like diet, exercise, genetic factors, comorbidities, and medications. Missing data on alcohol consumption and disease stage could also affect results. The lasso models used did not identify predictive variables, so no models were fitted.

Including both composite scores (MELD and FIB-4) and their components in models risks overfitting. Comparing models using the Bayes information criterion (BIC), those with individual components (bilirubin, INR, creatinine for MELD; age, AST, ALT, platelet count for FIB-4) had lower BIC. Thus, individual components were included in model selection. More models are needed for diverse populations.

BMI was excluded from models due to high rate of missingness (26%). Other measures had 16% missing values, estimated using multiple imputation using additive regression, bootstrapping, and predictive mean matching [7] with 3 burnins and 16 imputations. All intended modeling measures, including outcomes and complete laboratory data, were used for imputation. Patients could have multiple disease etiologies, so subtotals do not sum to the overall total.

In our study, albumin was inversely associated with adverse liver-related outcomes in cirrhosis patients. This aligns with the well-established evidence of the role of albumin in decompensated cirrhosis. In decompensated cirrhosis, serum albumin experiences structural and functional abnormalities, jeopardizing its antioxidant, scavenging, immune-modulating, and endothelium-protective functions. Consequently, the circulating pool of ‘effective’ albumin is substantially diminished, owing to both quantitative and qualitative alterations [9].

Our study has major limitations. It is a single center study, which may limit the generalizability of the findings. Our study was conducted at a tertiary care center with a liver transplant unit, enabling analysis of a large, well-characterized cohort. However, this setting may limit generalizability to community or secondary care populations, as patients often present with more advanced disease or complex comorbidities. Future studies should validate our models in diverse settings to ensure broader applicability. Diagnoses were based on ICD coding, which may lack accuracy and lead to coding errors and misclassification bias. While we applied strict inclusion criteria to minimize misclassification, the retrospective nature of the study limited our ability to systematically re-evaluate each patient’s diagnosis using gold-standard methods. Given the observational retrospective nature of the study, there is possibility for unmeasured confounding, despite multivariable adjustments for a large number of potential confounders. We did not have information on treatments for viral hepatitis or MASLD that our patient population may have received during the study period. Also, data on patients’ alcohol drinking habits were missing. Further, we did not have data on hepatitis B or C viral load suppression. Data on transjugular intrahepatic portosystemic shunt (TIPS) placement were not systematically captured in our dataset, as this information was not consistently documented in the EHR during the study period. While TIPS placement could influence outcomes, our study focused on identifying baseline clinical and laboratory variables associated with decompensation events. Our study categorized patients into distinct etiological groups (ALD, MASLD, viral hepatitis) based on primary documentation, but overlapping etiologies may still exist. This could influence the strength of associations, particularly in ALD, where confounding factors like lifestyle and comorbidities play a role. Future studies could explore models accounting for multiple contributing factors. The lack of predictive variables for ALD cirrhosis in our study may reflect the heterogeneity of this population and limitations in the data available for this group. Factors such as variability in disease severity, comorbidities, and lifestyle factors (e.g., alcohol use patterns, diet, and adherence to treatment) may have obscured the identification of consistent predictors. Additionally, our dataset lacked detailed information on alcohol consumption patterns and disease staging, which are critical for understanding the progression of ALD. Variables such as the duration and intensity of alcohol use, recent abstinence, nutritional status, and psychosocial determinants—including housing stability and continuity of care—are rarely captured in administrative datasets but may be essential for accurate risk stratification. Incorporating these elements into future models, potentially through linkage with clinical registries or prospective data collection, could enhance our ability to predict clinical deterioration in ALD. Future studies should aim to incorporate more granular data on these factors to improve risk prediction in ALD patients. Our study focused on developing associative models to estimate the short-term risk of decompensation events in cirrhosis, using baseline clinical and laboratory data. While competitive risk regression models are valuable for understanding long-term outcomes, they are less relevant for our study’s aim of providing practical tools for early risk stratification. Future studies could explore the integration of competitive risks, such as liver transplantation and death, to further refine prognostic models in cirrhosis. While the Youden index provides a balanced cut-off for risk prediction, a multilevel likelihood ratio approach could offer more nuanced risk stratification, particularly for patients at different stages of cirrhosis. Future studies could explore this approach to further refine prognostic accuracy. Our study did not adjust for hepatocellular carcinoma (HCC) as a time-dependent variable, as our primary focus was on developing associative models for short-term risk prediction using baseline clinical and laboratory data. While HCC is a critical determinant of prognosis in cirrhosis, its inclusion would require a different analytical approach, such as time-dependent Cox regression. Future studies should explore the integration of HCC as a time-dependent variable to further refine risk prediction models in cirrhosis. Lastly, we performed only internal validation of our new models. Nonetheless, our study has a number of strengths, including its relatively large sample size, categorization of cirrhosis groups by etiology of cirrhosis (ALD, viral hepatitis, and MASLD), evaluation of noninvasive tests that can be easily obtained during routine clinical practice, combining data from all health care settings (inpatient, outpatient), reliable baseline data and adjustment for multiple confounding factors. By not excluding patients with pre-existing decompensation outcomes, our models are designed to be ‘pre-predictive,’ identifying associations between baseline variables and the risk of subsequent adverse outcomes in a real-world patient population. This approach enhances the clinical utility of our models, as they can be applied to patients at various stages of disease progression. While the potential for synchronous effects (e.g., low platelets after a bleed being consequences rather than predictors) exists, the temporal separation between baseline measurements and outcomes helps mitigate concerns about reverse causality. To ensure the robustness of our models, we used five-fold cross-validation, which provides a rigorous assessment of model performance without requiring a separate validation cohort. While our models include variables used in MELD and Child-Pugh scores, their innovative contribution lies in their focus on short-term risk prediction for specific decompensation events and their etiology-specific design. This approach provides clinicians with a more targeted tool for early intervention and risk stratification. Our models provide valuable risk estimates that are applicable to the diverse and dynamic population of patients with cirrhosis. Our findings provide a foundation for developing risk stratification tools that integrate readily available clinical and laboratory data. These tools could be embedded in electronic health records to provide real-time risk estimates, enabling clinicians to identify high-risk patients early and tailor interventions accordingly. We acknowledge that, due to the lack of external validation, these models are not yet suitable for clinical use. Additional external validation studies are required to evaluate their generalizability beyond the single-center cohort used in this study. Future research should focus on validating these models in diverse populations, incorporating novel biomarkers, and exploring the integration of patient-reported outcomes and longitudinal data to further refine risk prediction in cirrhosis.

## 5. Conclusions

In summary, our new associative models provide accurate and consistent estimations of the risk of adverse liver-related outcomes in patients with cirrhosis using simple, routinely available baseline variables. These models could help identify high-risk patients, prompting timely interventions and targeted treatment strategies. Further studies are needed to assess their generalizability before they can be confidently used as clinical decision tools in practice.

## Supporting information

Supplementary Table 1 ICD 9 and 10 codes for etiologies of chronic liver disease and cirrhosis outcomes. (DOCX)
