# Supplementary material for: Liver-related outcomes in patients with cirrhosis: The value of clinical and laboratory data and noninvasive tests
Source: PLoS One. 2025 Jul 1;20(7):e0326702. doi: 10.1371/journal.pone.0326702 (PMC12212549; doi:10.1371/journal.pone.0326702)
Supplement: Supplementary Table 1 — (DOCX) [file pone.0326702.s001.docx]

**SUPPLEMENTARY MATERIAL**

**Supplementary Table 1: ICD 9 and 10 codes for etiologies of chronic liver disease and cirrhosis outcomes.**

**Cirrhosis Etiology:**

571.8 other chronic nonalcoholic liver disease

571.9 unspecified chronic liver disease without alcohol

571.5 - Cirrhosis of liver without mention of alcohol

K75.81 nonalcoholic steatohepatitis

K76.0 fatty liver, NOS

571.0 alcoholic fatty liver disease

571.1 acute alcoholic hepatitis

571.2 alcoholic cirrhosis of liver

571.3 alcoholic liver damage

K70.10-11 alcoholic hepatitis/liver disease

F10 alcohol dependence/abuse

070.0-9 viral hepatitis A, B, C, D, E and unspecified

V02.60-69 carrier or suspected carrier of viral hepatitis

571.40-41 chronic hepatitis unspecified

571.42 autoimmune hepatitis

571.49 chronic hepatitis

573.1-2 hepatitis in viral diseases classified elsewhere

B15.0-B19.9 Hepatitis A, B and C

Z22.50-59 Carrier of viral hepatitis

K71.2-6 Toxic liver disease

K73.0-9 Chronic hepatitis

K75.2-4 Autoimmune hepatitis

O98 Viral hepatitis complicating pregnancy

275.01 hemochromatosis

E83.11 hemochromatosis

E88.01 Alpha-1-antitrypsin deficiency

E83.01 Wilson’s disease

**Cirrhosis Outcomes:**

| ***Complication*** | ***ICD 9*** | ***ICD 10*** |
| --- | --- | --- |
| Ascites | 789.5 (789.51-789.59) | R18 (R18.0-18.8) |
| Hepatic Encephalopathy | 572.2 | K72.91  K72 (K72.00, K72.01, K72.10, K72.11, K72.90, K72.91) |
| Varices | 456.21, 456.1, 456.8 | I85.00, I86.4 |
| Variceal bleeding | 456.0  530.82  578 | I85.01  I85.11 |
